# Effects of dexmedetomidine on the deformability of erythrocytes *in vitro* and in anesthesia

**DOI:** 10.3892/etm.2014.1633

**Published:** 2014-03-27

**Authors:** XIAO-MING YANG, JUN LIU, JUN JI, JIE XIE

**Affiliations:** 1 Department of Anesthesiology, Air Force General Hospital, PLA, Beijing 100142, P.R. China; 2 Jiangsu Province Key Laboratory of Anesthesiology, Xuzhou Medical College, Xuzhou, Jiangsu 221002, P.R. China; 3 Jiangsu Province Key Laboratory of Anesthesiology and Analgesia Application Technology, Xuzhou, Jiangsu 221002, P.R. China; 4 Department of Information, Air Force General Hospital, PLA, Beijing 100142, P.R. China

**Keywords:** dexmedetomidine, erythrocyte deformability, nitric oxide, endothelial nitric oxide synthase, cholecystectomy, laparoscopic

## Abstract

The current study aimed to evaluate the impact of clinically relevant concentrations of dexmedetomidine on the deformability of erythrocytes *in vitro* and the effects of dexmedetomidine on the deformability of erythrocytes in patients undergoing laparoscopic cholecystectomy. Erythrocyte suspensions of different concentrations were divided into six groups: Control (group C); low, medium and high concentrations of dexmedetomidine (groups DL, DM and DH, respectively); yohimbine alone (group Y) and yohimbine mixed with dexmedetomidine (group YD). The suspensions were incubated in a thermostatic shaking incubator (50 rpm, 37°C) for 60 min. The nitric oxide (NO) concentrations and endothelial nitric oxide synthase (eNOS) activities of red blood cells and the erythrocyte deformability index (EI) were then measured. Patients (n=40) scheduled for laparoscopic cholecystectomy were randomly divided into a dexmedetomidine group (group A) and a control group (group B). The induction and maintenance of anesthesia in the two groups was identical. The EI and hematocrit (Hct) were assayed prior to anesthesia (T_0_) and following the surgery (T_1_). In the *in vitro* assay, the EI, the activity of eNOS and the NO concentration of the erythrocytes were significantly higher in groups DL, DM, DH and YD than in group C (P<0.05). In addition, the EI, the eNOS activity and NO concentration of the erythrocytes were higher in group DM than in group YD (P<0.05). In the patients, the EI value at T_1_ (0.90±0.04) was higher than at T_0_ (0.81±0.06) in group B (P<0.05). No statistically significant difference between the EI values at T_0_ and T_1_ was identified in group A (P>0.05). Dexmedetomidine treatment is able to improve the deformability of erythrocytes *in vitro* and in anesthesia. The improvement of erythrocyte deformability by dexmedetomidine may be partially associated with adrenergic receptors through activation of eNOS to enhance the concentration of NO in red blood cells.

## Introduction

Anesthesia, surgery, stress, intraoperative blood transfusion, the ambient temperature and other factors may cause changes in perioperative blood rheology (1–3). Moreover, perioperative changes in blood rheology are closely associated with postoperative venous thrombosis, microcirculation dysfunction and postoperative complications such as infection (4,5). Erythrocyte deformability means that red blood cells change their morphological characteristics under the action of external forces. Good red blood cell deformability is the basis for ensuring effective microcirculatory perfusion and normal physiological function. Due to the application of CO_2_ pneumoperitoneum during laparoscopic surgery, the decreased pH of patient serum may slow down blood flow, increase resistance, decrease the ability to transport oxygen, lower microcirculation hypoperfusion and impair red blood cell function (6). Therefore, drugs that improve erythrocyte deformability during anesthesia and surgery are able to treat and prevent the development of intraoperative and postoperative complications in patients.

Dexmedetomidine is a highly selective α_2_ adrenoceptor agonist. It has the characteristics of a sedative, analgesic and anti-shivering, has little effect on hemodynamics and does not cause respiratory depression. Hence, it is widely used in intensive care units (7). Dexmedetomidine may spare the use of opioids and anesthetics in surgery, mediate feedback inhibition of norepinephrine release and protect important organs against ischemia-reperfusion injury (8–10). A recent study has shown that dexmedetomidine protects erythrocytes against the changes in deformability induced by hepatic ischemia-reperfusion injury in rats (11).

In the present study, the effects of dexmedetomidine on isolated human erythrocytes were investigated and the protective effects of dexmedetomidine on erythrocytes in patients a stressed environment during laparoscopic cholecystectomy were observed.

## Materials and methods

### Erythrocytes in vitro

Whole blood samples (5 ml) were centrifuged at 1,500 × g to separate the erythrocytes. Subsequently, the erythrocytes were washed three times in phosphate-buffered saline (PBS). An erythrocyte suspension with a concentration of 2% was prepared by adding 1 ml erythrocytes to 49 ml PBS. The erythrocyte suspension was divided into six groups: Control group (group C); low concentration dexmedetomidine (Jiangsu Hengrui Medicine Co. Ltd. Jiangsu, China) group (group DL); medium concentration of dexmedetomidine (group DM); high concentration of dexmedetomidine (group DH); yohimbine (Sigma Chemical Co., St. Louis, MO, USA) alone group (group Y) and yohimbine plus dexmedetomidine group (group YD). In each group, 2 ml samples of erythrocyte suspension were tested. Dexmedetomidine was added to the suspensions of the DL, DM and DH groups with final concentrations of 0.6, 1.8 and 5.4 ng/ml, respectively. Yohimbine was added to the suspension of group Y with a final concentration of 0.1 *μ*mol/l. Yohimbine and dexmedetomidine were added to the suspension of group YD with final concentrations of 0.1 *μ*mol/l (yohimbine) and 1.8 ng/ml (dexmedetomidine). The six groups of suspensions were placed into a thermostatic concussion incubator (50 rpm, 37°C; Sihuan Scientific Instrument Factory, Beijing, China) for 60 min.

This study used a laser diffraction method to measure the deformability of the red blood cells under different shear forces with a LBY-BX erythrocyte deformability instrument (Plymouth Health Instruments Co., Ltd. Beijing, China). The test samples were added to 37°C isotonic polyvinylpyrrolidone (PVP) and measured twice when H values for each sample were within a range of 12 to 16%. The erythrocyte deformation index (EI) was used as an indicator of erythrocyte deformation. Nitrate reductase was used to determine the nitric oxide (NO) concentration in the red blood cells and an ELISA kit was used to measure endothelial nitric oxide synthase (eNOS) activities of erythrocytes. Kits were purchased from Beijing Huaying Biotechnology Co. (Beijing, China).

### Clinical investigation

This study was conducted in accordance with the Declaration of Helsinki and with approval from the Ethics Committee of Air Force General Hospital, PLA (Beijing, China). Written informed consent was obtained from all patients included in the study. The study population comprised 40 patients aged 18–60 years, with American Society of Anesthesiologists (ASA) status 1 or 2, undergoing laparoscopic cholecystectomy at Air Force General Hospital. Patients who were older than 60 years; had a history of psychiatric and/or neurological illness; had cardiovascular disease; were hypertensive; were morbidly obese; had a known allergic reaction to any of the study medication; had recently been exposed to sedatives or analgesics; or had significant laboratory abnormalities were excluded. The 40 patients were randomly divided into two groups: Dexmedetomidine group (group A) and control group (group B). Heart rate (HR), blood pressure (BP), pulse oxygen saturation (SpO_2_), electrocardiogram (ECG), respiratory end-tidal CO_2_ pressure (PetCO_2_) and other vital signs of the patients were routinely monitored after they entered the surgery. Prior to being submitted, the patients in group A received an intravenous (IV) infusion of dexmedetomidine (2 ml diluted in 48 ml saline) at a dose of 0.5 μg/kg over 10 min using an infusion pump. After pre-oxygenation for 10 min, general anesthesia was induced with midazolam 0.03–0.05 mg/kg, sufentanil 0.2–0.5 μg/kg and propofol 1.5–2.5 mg/kg through slow IV administration. Endotracheal intubation was facilitated with cisatracurium 0.15 mg/kg IV. Anesthesia was maintained until the termination of pneumoperitoneum by the administration of propofol 4–8 mg/kg/h, remifentanil 0.1–2 μg/kg/h, cisatracurium (a third to a half of the initial dose once every 40 min) and dexmedetomidine (0.5 μg/kg/h) infusion. The patients in group B received the same anesthetic as those in group A, but were administered normal saline instead of the dexmedetomidine IV infusion. The anesthesiologist was permitted to treat hemodynamic events. Reversal of neuromuscular blockade was achieved with neostigmine 0.05 mg/kg and glycopyrrolate 0.008 mg/kg through slow IV administration. Tracheal extubation was carried out when adequate muscle tone was achieved and respiration was satisfactory.

The EI and hematocrit (Hct) were evaluated prior to anesthesia (T_0_) and immediately after the surgery (T_1_). Hct was measured with a full-automatic biochemistry analysis meter (Tainuo Science And Trade Co. Ltd., Shandong, China) To determine the EI, 5 ml blood was taken from an upper extremity opposite to that of the site of intravenous administration at T_0_ and T_1_ respectively. Changes in the EI in flowing blood were measured using the ZL9000 hemorheology instrument (Zhongchi Weiye Technology Development Co., Ltd., Beijing, China).

### Statistical analysis

The results were expressed as mean ± standard deviation. Data were compared using a t-test and ANOVA (one-way ANOVA). Data between groups was compared using a q-test. Statistical analysis was performed with SPSS statistical software, version 13.0 (SPSS, Inc., Chicago, IL, USA). P<0.05 was considered to indicate a statistically significant difference.

## Results

### Erythrocytes in vitro

The values of the EI were used to measure the deformation of red blood cells, which was determined by a laser diffraction method. A larger EI value was indicative of a greater erythrocyte deformability. The EI value, NO levels and eNOS activity in erythrocytes were higher in the DM group than in all other groups. The EI values, concentration of NO and activity of eNOS in erythrocytes were significantly higher in groups DL, DM, DH and YD than in group C (P<0.05). No significant difference was identified in these variables between group Y and group C (P>0.05). The EI values, concentration of NO and activity of eNOS in erythrocytes were higher in group DM compared with the respective values in group YD (P<0.05; Table I).

### Clinical investigation

In the clinical analysis, the values of EI were evaluated by measurements of viscosity, where a larger EI value was indicative of poorer erythrocyte deformability. The Hct and EI values at T_1_ and T_0_ revealed no statistically significant differences between groups A and B. The EI values at T_1_ and T_0_ respectively revealed no statistically significant differences between groups A and B (P>0.05). The EI at T_1_ (0.90±0.04) was higher than that at T_0_ (0.81±0.06) in group B (P<0.05). No statistically significant differences were identified in Hct and EI between T_0_ and T_1_ in group A (P>0.05; Table II).

## Discussion

The results of the *in vitro* assay showed that low, medium and high concentrations of dexmedetomidine are able to elevate the NO levels and eNOS activity in red blood cells, indicating that dexmedetomidine treatment directly improves the deformability of red blood cells. Yohimbine, which is an α_2_ adrenergic receptor antagonist, had no effect on red blood cell deformability when used alone. The deformability of erythrocytes in group YD, which was treated with dexmedetomidine and yohimbine, was increased when compared with that in the control group, and decreased when compared with that in group DM. This indicates that yohimbine antagonized the dexmedetomidine-induced improvements of red blood cell deformability.

A previous study has demonstrated that the hemorheology of patients undergoing laparoscopic cholecystectomy following pneumoperitoneum is significantly changed compared with the preoperative hemorheology (12). This is the reason for using patients undergoing laparoscopic cholecystectomy as research subjects in the current study. The value of EI was affected by the Hct measured by the viscosity method (13). In the clinical experiment of the present study, Hct did not change significantly prior to and following surgery (P>0.05) in the two groups of patients. This indicated that in the present study, Hct had no impact on EI. In group B, EI was significantly higher in patients at T_1_ compared with that at T_0_ (P<0.05), demonstrating that erythrocyte deformability in patients was decreased following surgery. In group A, EI was higher in patients at T_1_ compared with that at T_0_; however, the increase was not significant (P>0.05). This suggests that the perioperative use of dexmedetomidine is able to improve erythrocyte deformability impaired by surgery, anesthesia, stress and other adverse effects, and maintain stable perioperative blood rheology. Animal studies conducted by Arslan *et al* showed that dexmedetomidine improved erythrocyte deformability, which is consistent with the results of the present study (11).

NO molecules *in vivo* are active free radicals and have a role as cell signaling molecules, with a wide range of physiological effects. Their cardiovascular activities are particularly important. The concentration of NO in erythrocytes has an important role in maintaining erythrocyte deformability and regulating red blood cell deformability. Appropriate concentrations of NO allow erythrocytes to reach maximum deformability, while an excess of NO exhibits free radical characteristics and therefore damages red blood cell deformability (14–16). Studies have demonstrated that NO combines with hemoglobin β^93^Cys residues to form S-nitrosohemoglobin, and that the S-nitrosohemoglobin in cytoskeletal proteins may increase red blood cell deformability (17,18). Studies in healthy volunteers have indicated that dexmedetomidine at clinically relevant doses acts on the α_2_ adrenergic receptors of endothelial cells, thereby activating eNOS and increasing the body’s NO levels (14,19).

A variety of red blood cell membrane receptors have been reported since 1992, including α_1_ adrenergic receptors and β-adrenergic receptors (20,21). Although the existence of an α_2_ adrenoceptor in red blood cells has, to the best of our knowledge, not yet been reported in literature, the results of the current study could be used to support it. Yohimbine antagonized the dexmedetomidine-induced improvements in erythrocyte deformability, suggesting that red blood cell membranes may present α_2_ adrenergic receptors. However, whether the mechanism by which dexmedetomidine affects erythrocyte deformability depends on the existence of α_2_ adrenergic receptors on the erythrocyte membrane is not clear and remains to be studied further.

Anesthesia during surgery, due to preoperative underlying diseases, the use of a variety of vasoactive and narcotic drugs, blood transfusion, the ambient temperature and other factors, may lead to changes in hemorheology and microcirculation in the body. This significantly reduces the tolerance of the body to surgery and extends postoperative recovery. However, studies have revealed that drugs, such as desflurane, sevoflurane and propofol, affect the deformability of red blood cells (22–24). Therefore, using drugs that increase red blood cell deformability during perioperative anesthesia has an important clinical significance.

In summary, dexmedetomidine treatment increased the deformability of red blood cells *in vitro*. Perioperative use of dexmedetomidine (0.5 μg/kg infused over 10 min, followed by a maintained dose of 0.5 μg/kg/h until the end of pneumoperitoneum) significantly improved the erythrocyte deformability of patients following laparoscopic cholecystectomy. This may be related to the effect of dexmedetomidine on erythrocyte eNOS, which leads to an increased NO concentration in red blood cells.

## Figures and Tables

**Table I:** EI values, NO levels and eNOS activity in erythrocytes after incubation of the red cell suspension for 60 min (n=9, mean ± SD).

Groups	EI (%)	NO (*μ*mol/gHB)	eNOS (U/mg HB)
C	21.01±1.48	4.136±0.322	21.096±2.532
DL	23.58±1.99^a^	5.036±0.835^a^	23.893±2.393^a^
DM	25.92±1.67^a^,^b^	5.442±0.847^a^,^b^	25.911±2.442^a^,^b^
DH	23.26±1.74^a^	5.139±0.969^a^	24.922±4.077^a^
Y	22.02±2.47	4.137±0.679	22.264±2.631
YD	24.02±1.61^a^	4.648±0.715^a^	23.636±1.861^a^

a P<0.05 compared with group C;

b P<0.05 compared with group YD.

Group C, control; group DL, low concentration of dexmedetomidine; group DM, medium concentration of dexmedetomidine; group DH, high concentration of dexmedotomidine; group Y, yohimbine; group YD, yohimbine and medium concentration of dexmedetomidine. EI, erythrocyte deformability index; NO, nitic oxide; eNOS, endothelial nitric oxide synthase.

**Table II:** Values of EI and Hct in two groups of patients at T_0_ and T_1_ (n=20, mean ± SD).

	EI (%)		Hct (l/l)	
		
Groups	T_0_	T_1_	T_0_	T_1_
B	0.81±0.06	0.90±0.04^a^	0.37±0.03	0.36±0.04
A	0.82±0.07	0.85±0.06	0.38±0.04	0.37±0.04

a P<0.05 compared with T_0_ in group B.

Group B, control; Group A, dexmedetomidine treatment group; T_0_. prior to treatment; T_1_, immediately after surgery. EI, erythrocyte deformability index; Hct, hematocrit.
